# The interaction network between group 3 innate lymphoid cells and other cells

**DOI:** 10.1016/j.fmre.2023.10.021

**Published:** 2023-12-30

**Authors:** Yi-tong Hu, Xing-zi Liu, Yue-miao Zhang, Xiaohuan Guo

**Affiliations:** a Renal Division, Department of Medicine, Peking University First Hospital, Beijing 100034, China; b Institute of Nephrology, Peking University, Beijing 100034, China; c Key Laboratory of Renal Disease, Ministry of Health of China, Beijing 100034, China; d Key Laboratory of Chronic Kidney Disease Prevention and Treatment (Peking University), Ministry of Education, Beijing 100034, China; e Research Units of Diagnosis and Treatment of Immune-mediated Kidney Diseases, Chinese Academy of Medical Sciences, Beijing 100034, China; f Peking University Health Science Center, Beijing 100191, China; g Institute for Immunology, Tsinghua University, Beijing 100084, China; h Department of Basic Medical Sciences, School of Medicine, Tsinghua University, Beijing 100084, China; i Beijing Key Lab for Immunological Research on Chronic Diseases, Tsinghua University, Beijing 100084, China

**Keywords:** Group 3 innate lymphoid cells, Cell-cell interactions, Innate immune cells, Adaptive immunity, Epithelial cells, Interleukin-22

## Abstract

Group 3 innate lymphoid cells (ILC3s) have emerged as significant regulators of tissue homeostasis, immunity and inflammation through various effector molecules in response to signals within the tissue microenvironment. Multiple signals derived from the tissue microenvironment have been illustrated in recent years, which could either activate or suppress the activity of ILC3s. ILC3s, in turn, could also influence many other cells through their effector molecules, such as interleukin (IL)-22, IL-17 and lymphotoxins, indicating that ILC3s act as an important hub in the complex network of cell interactions. In this review, we discuss our current appreciation of the functional crosstalk between ILC3s and myeloid cells, T and B cells, epithelial cells or other types of cells, thus highlighting the emerging importance of communication between these cells for the prevention or induction of relevant diseases and providing insights for future directions of investigation and therapeutic interventions.

## 1 Introduction

Over the past decade, we have witnessed rapidly emerging and exciting advances in the understanding of a novel family of leukocytes termed innate lymphoid cells (ILCs), which lack the expression of recombination activating gene (RAG)-dependent rearranged antigen receptors but have substantial similarities to T helper (Th) cells [1,2]. Similar to T cells, ILCs also initially originate from common lymphoid progenitors in the fetal liver and bone marrow later in the adult [3]. ILCs can be further subdivided into three distant groups in view of the cytokines that they produce and the transcription factors that regulate their development and function [1]. Group 1 ILCs (ILC1s), which comprise cytotoxic conventional NK cells and noncytotoxic ILC1s, express the T-box transcription factor T-bet and produce the cytokine interferon-γ (IFN-γ). Group 2 ILCs (ILC2s) are GATA3- and RORα-dependent, and produce Th2 cell-associated cytokines including interleukin (IL)-5 and IL-13. Group 3 ILCs (ILC3s) produce IL-22 and/or IL-17A, and require RORγt for their development and function, consisting of lymphoid tissue inducer (LTi) or LTi-like cells, natural cytotoxicity receptor (NCR)^+^ ILC3s and NCR^−^ ILC3s [1]. LTi cells express c-Kit and CCR6, but not NCR. They play important roles in the formation of secondary lymph nodes and Peyer's patches at the fetal stage through lymphotoxin (LT)-β receptor (LTβR) signaling [2]. NCR^+^ and NCR^−^ ILC3s are distinguished by the expression of the NCR NKp46 in mice or NKp44, NKp46 and NKp30 in humans [4]. Diffusely localized in the intestine lamina propria in mice, NKp46^+^ ILC3s can secrete IL-22 and IFN-γ but not IL-17A, whereas NKp46^−^ ILC3s produce both IFN-γ and IL-17A but less IL-22 [1,2,5,6]. Human NKp44^+^ ILC3s, mostly co-expressing NKp46, constitute the majority in adult barrier tissues such as the gut, tonsil, uterus, and skin during homeostasis and represent the primary source of IL-22, but not produce IL-17A [1,5,7]. NKp44^−^ ILC3s are the predominant population in secondary lymphoid organs, skin, adipose tissue, and lung, expressing mostly IL-17 transcripts [1,7]. Contrary to mouse ILC3s, *ex-vivo*-isolated human ILC3s, regardless of their NKp44 expression, do not express IFN-γ [2,5].

ILC3s functionally mirror CD4^+^ Th17 cells [2]. In contrast to T and B cells, ILC3s do not recognize the specific antigen through RAG-dependent rearranged antigen receptors, but are instead activated rapidly by proinflammatory cytokines released from other cells like macrophages, dendritic cells (DCs), T and B cells, and epithelial cells in the tissue microenvironment. Subsequently, ILC3s produce multiple effector molecules and influence the survival, proliferation, migration or functions of these cells to regulate the tissue homeostasis, host immunity or inflammation [8]. Thus, there is a complex interplay network centered around ILC3s. Here, we summarize our current understanding of the functional interactions between ILC3s and other cells, discuss the limitations and future areas of investigation, and consider the potential for these interactions to be therapeutic interventions to benefit human health.

## 2 Crosstalk between ILC3s and epithelial cells

ILC3s are critical to maintain the integrity and function of barrier tissues such as the skin, intestine, and lung by promoting epithelial cell proliferation and provoking immune responses. The receptors of ILC3 effector cytokines, such as IL-22 and IL-17, play key roles in the crosstalk between ILC3s and epithelial cells. Here we summarize the interactions between ILC3s and intestinal epithelial cells (IECs), keratinocytes (KCs), lung epithelial cells (LECs) and other epithelial cells.

### 2.1 ILC3s and IECs

The intestine is the major harbor of ILC3s. ILC3s produce multiple effector molecules to regulate the IEC function. Among them, IL-22 functions as an important mediator of the crosstalk between ILC3s and IECs under steady-state and infection conditions. ILC3-derived IL-22 can facilitate intestinal epithelial restitution after tissue destruction via inducing cell proliferation and maintaining intestinal epithelial stem cells (ISCs), which might be involved in injury reduction of colitis and other intestinal inflammatory diseases [9], [10], [11]. ISCs have been found to express IL-22 receptor (IL-22R), through which IL-22 protects ISCs against inflammatory tissue damage mediated by graft versus host disease (GVHD) [12]. Moreover, a recent study showed that IL-22 signaling in colon epithelial cells had a significant role in protecting them against tumor development by curtailing mutations in ISCs, thus providing adjunctive therapeutic interventions for avoiding genotoxic sequelae after a variety of clinical therapies such as chemotherapy, radiotherapy and bone marrow transplantation [13]. In addition to ISCs, IL-22 promotes IECs expressing antibacterial peptides such as RegIIIβ and RegIIIγ, which may have functions of directly killing bacteria, preventing the invasion of *Citrobacter rodentium* (*C. rodentium)* deep into the colonic crypts, and promoting epithelial repair. Besides, IL-22 and its downstream effectors may also protect the intestinal integrity by regulating the tight junctions as well as the permeability of colonic epithelial cells [14]. Therefore, IL-22 plays a protective role in maintaining the intestinal barrier integrity in response to tissue insult and microorganism invasion. IL-22 can also activate Fut2 expression in IECs, which specifically regulates α1,2-Fucose on enterocytes and goblet cells that mediate interplay between IECs and luminal microorganisms to maintain homeostatic intestinal microenvironment as well as to defend against pathogenic bacteria [15]. Converse to its protective effects, IL-22 also promotes the expression of proinflammatory cytokines tumor necrosis factor (TNF)-α and IL-8 in IECs and these cytokines in turn increase IL-22R1 expression, which may further strengthen the proinflammatory effect of IL-22 in inducing Crohn's disease [9]. In addition, the roles of ILC3s within tumors may also not always be desirable. ILC3-produced IL-22 may induce abnormal proliferation of IECs and thus function in the transition from chronic inflammation to colon cancer as well as the perpetuation of cancer [16].

Besides its effects on IECs and ISCs, IL-22 facilitates the production of mucus-associated protein (Muc) by goblet cells via induction of signal transducer and activator of transcription 3 (STAT3), thereby promoting the formation of mucus layer and attenuating chronic colitis. Consistently, the spontaneous recovery of acute colitis is accompanied by upregulation in IL-22 expression, which leads to the restitution of goblet cells [17]. In addition, similar to IECs, Paneth cells can also be induced by IL-22 to derive S100A8, S100A9, RegIIIβ and RegIIIγ to defend against microbial invasion [18].

Apart from IL-22, IL-17 may also mediate the interactions between ILC3s and IECs. In stimulation with IL-23, ILC3s (Thy1^high^SCA-1^+^ ILCs) can secrete IL-17 and IFN-γ, both of which have essential roles in innate immune-mediated colitis induced by *Helicobacter hepaticus* [19]. Besides, IL-23-induced IL-17A production by ILC3s can drive inflammatory bowel disease (IBD) in *Tbx21^−/^*^−^ *Rag2^−/−^* ulcerative colitis (TRUC) mice [20]. These observations provide potentially relevant insights into IL-17 blockade for the treatment of human IBD.

In addition to IL-22 and IL-17, ILC3-derived lymphotoxins (LTs) can also mediate interactions between ILC3s and IECs. Like IL-22, LTα is also involved in the induction and maintenance of Fut2 expression, and Fut2 can subsequently catalyze epithelial fucosylation in the gastrointestinal tract, which has a role in preventing bacterial infection [21]. Nevertheless, contrary to the production of IL-22, which is mediated by commensal bacteria, LTs expression is commensal flora-independent [21]. Moreover, it has been shown that ILC3-expressed LTs can stimulate LTβR signaling in IECs and subsequently recruit neutrophils to the gut through upregulating CXCL1 and CXCL2 expression, thus also protecting against mucosal bacterial infection [22].

IECs are also critical to the regulation of ILC3 responses. Apart from IL-22R, IECs also express IL-22 binding protein (IL-22BP), indicating that they can potentially suppress excessive and pathologic activity of IL-22 signaling [9,13]. An early study revealed that the effect of IL-22 on IECs can be suppressed in the presence of IL-22BP [23]. Moreover, the microbiota residing in the gut repress IL-22 production in ILC3s through the expression of IL-25 by epithelial cells [24]. Critically, the inhibitory effect of IL-25 on ILC3s is indirect due to the lack of IL-25 receptor expression by ILC3s and is considered to be mediated by contact between IL-17RB^+^ ILCs or DCs and ILC3s [24]. Furthermore, IEC-intrinsic inhibitor of κB kinase α (IKKα) expression can maintain ILC3-mediated antibacterial immunity in the intestine potentially by restraining the thymic stromal lymphopoietin (TSLP) expression by IECs, which can impair IL-22 production by ILC3s and thus suppress innate immunity to *C. rodentium* [25]. Converse to IL-25 and IL-22BP, IL-1α released by rotavirus-infected IECs potently promotes ILC3 production of IL-22, which in turn acts in synergy with IEC-derived IFN-λ on IECs to trigger STAT1 signaling and augment expression of interferon-stimulated genes for protection against viral infection [26]. Such cooperation between these two cytokines might be harnessed for the clinical therapy of chronic viral infection [26]. In addition, LTβR signaling can induce IL-23 production by IECs and further drive IL-22 secretion by CD4^−^ LTi cells, thereby promoting the mucosal wound healing following epithelial injury [27].

**Fig. 1:**
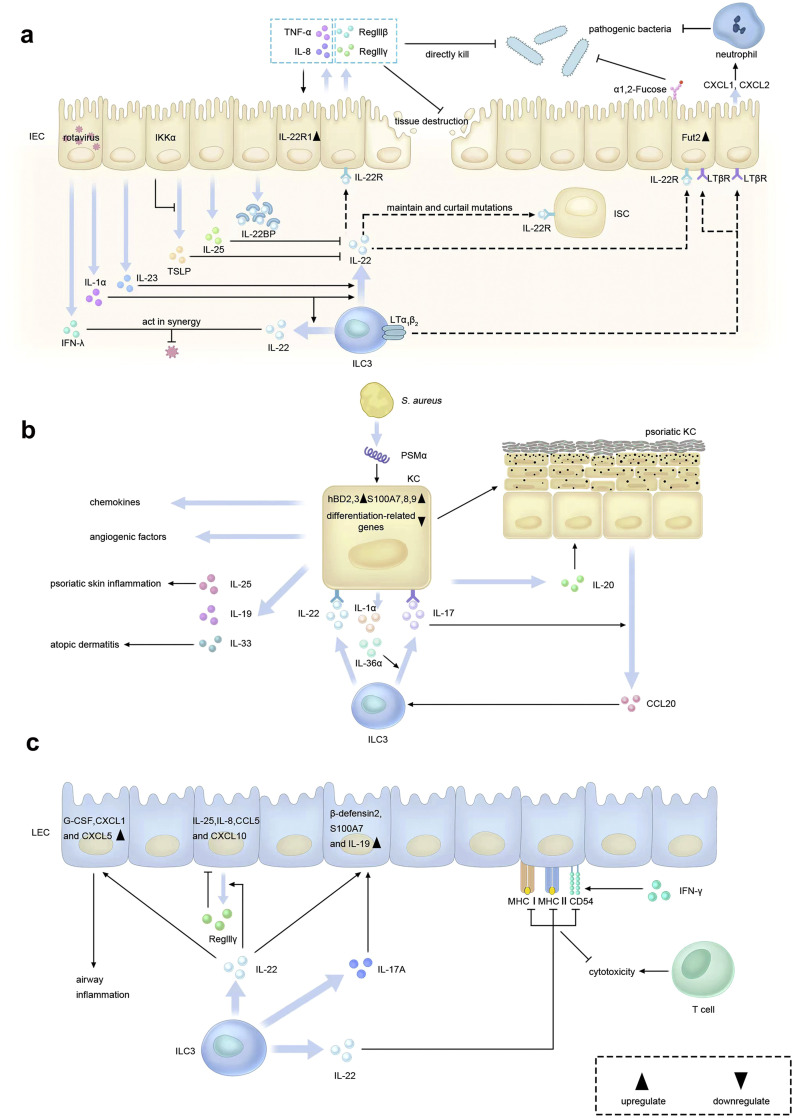
**Interactions between ILC3s and epithelial cells.** (a) Crosstalk between ILC3s and IECs. ILC3-produced IL-22 and LTs induce multiple effector molecules in IECs, which may protect against bacterial infection and tissue destruction. Additionally, IL-22 can maintain and curtail mutations in ISCs via IL-22R. IECs can also inhibit IL-22 signaling by releasing IL-22BP, IL-25 and TSLP, whilst promoting ILC3 secreting IL-22 by IL-1α and IL-23. (b) Crosstalk between ILC3s and KCs. ILC3-derived IL-22 and IL-17 can increase the expression of inflammatory skin disease-associated molecules in KCs. KC-derived IL-1α, IL-36α and CCL20 can regulate or recruit ILC3s. (c) Crosstalk between ILC3s and LECs. ILC3-derived IL-22 can promote the expression of anti-microbial peptides in airway epithelial cells, and also upregulate G-CSF, CXCL1 and CXCL5 for airway inflammation. Moreover, IL-22 can inhibit IFN-γ-induced MHC upregulation on HBE cells and thus defend against T cell-mediated cytotoxicity. Abbreviations: ILC3s, group 3 innate lymphoid cells; IECs, intestinal epithelial cells; IL, interleukin; IL-22R, IL-22 receptor; LT, lymphotoxin; ISCs, intestinal epithelial stem cells; IL-22BP, IL-22 binding protein; TSLP, thymic stromal lymphopoietin; IKK, inhibitor of κB kinase; KCs, keratinocytes; hBD, human β-defensin; *S. aureus, Staphylococcus aureus*; PSM, phenol-soluble modulin; LECs, lung epithelial cells; HBE, human bronchial epithelial; MHC, major histocompatibility complex; G-CSF, granulocyte colony-stimulating factor.

Collectively, there are intensive interactions between ILC3s and IECs, and IL-22 has a pivotal role in modulating these interactions (Fig. 1a). Controlling ILC3 production of IL-22 might be critical to designing future therapeutic strategies for preventing infectious diseases and minimizing intestinal damage in patients.

### 2.2 ILC3s and KCs

ILC3s are found in the skin tissue and regulate the skin barrier function. Evidence has demonstrated that ILC3s are implicated in several skin-related diseases. An increased number of ILC3s has been found in skin and peripheral blood of patients with psoriasis, and IL-22 protein concentrations are also significantly higher in the blood plasma from psoriasis patients compared to the levels of healthy participants and positively correlate with the disease severity [28,29]. ILC3-derived IL-22 triggers phosphorylation of STAT3 in KCs via IL-22R and upregulates the expression of human β-defensin (hBD) 2 and 3, which is similar to its function in IECs and also confers protection against pathogens [30,31]. Importantly, the increased levels of hBDs may be associated with inflammatory skin diseases such as psoriasis or atopic dermatitis [30]. Moreover, IL-22 significantly increases the expression of proinflammatory molecules S100A7, S100A8 and S100A9, which are expressed at low or undetectable levels in normal epidermis and nondifferentiated cultured KCs but are high in abnormally differentiated psoriatic KCs [31]. IL-22-induced S100A8, S100A9 and matrix metalloproteinase 3 also have a significant role in inducing KC migration to the injury site to promote wound healing [31]. In addition, KC differentiation can be inhibited by IL-22 via downregulation of differentiation-related genes, resulting in hyperplasia of the KC layers and psoriasis-like epidermal alterations, including acanthosis, loss of granular layer, and a compact cornified layer [31,32]. Furthermore, IL-22 potently induces the production of chemokines and angiogenic factors in KCs, which play crucial roles in recruiting leukocytes and promoting angiogenesis in the skin respectively, thus further promoting the inflammatory response [33]. Notably, IL-22 induces IL-20 production in KCs, which is also a key mediator of the epidermal alteration in psoriasis, and exerts very similar effects on KCs as IL-22 does, partially mediating certain effects of IL-22 on KCs [32,34]. These findings suggest that ILC3s and IL-22 might play important roles in the pathogenesis of psoriasis comprising substantial epidermal swelling, abnormal KC differentiation and inflammatory cell infiltration.

In addition to IL-22, ILC3-produced IL-17 could be associated with skin pathology in psoriasis [35,36]. Like IL-22, IL-17 also activates the production of antimicrobial peptides like S100A7 and hBDs, proinflammatory cytokines and chemokines in KCs [28]. In detail, IL-17 promotes IL-20 expression in KCs, synergizes with IL-22 in triggering chemokines in KCs and further amplifies the IL-22-induced production of IL-20 in KCs [34]. In particular, IL-17 can also promote the expression of the pro-proliferative cytokine IL-19 in KCs [28]. Moreover, IL-25 production in KCs can be significantly induced by IL-17 and promotes the expression of proliferation and pro-inflammatory genes in KCs, thus contributing to the pathogenesis of psoriatic skin inflammation [37]. In addition to psoriasis, ILC3-produced IL-17A also increases IL-33 secretion from KCs, which induces type 2 immune responses and leads to the progression of atopic dermatitis [38].

KCs can also activate ILC3s and thus cause inflammatory skin diseases. It has been reported that KCs stimulated by *Staphylococcus aureus*-produced phenol-soluble modulin α can release IL-1α and IL-36α to trigger the production of IL-17 from ILC3s and subsequently lead to skin inflammation [39]. Moreover, psoriatic KC-produced CCL20 functions as a potent chemokine for ILC3s, and ILC3-derived IL-17A can in turn facilitate the release of critical cytochemokines like CXCL1, CXCL2 and CCL20 by KCs, thereby forming positive feedback and worsening skin inflammation [40].

Collectively, the crosstalk between ILC3s and KCs plays an important role in inflammatory skin disorders like psoriasis and atopic dermatitis, thus providing an unprecedented opportunity to therapeutically target or harness the interactions to treat these diseases (Fig. 1b).

### 2.3 ILC3s and LECs

The presence of ILC3s in human lung has also been demonstrated [41]. IL-22 predominantly derived from ILC3s is critical to the crosstalk between ILC3s and LECs [42] (Fig. 1c). Like the effects of IL-22 on KCs, it can also induce STAT3 phosphorylation in LECs via IL-22R1 and facilitate LECs proliferation [42], [43], [44]. Interestingly, IL-22 seems to have both protective and proinflammatory effects on LECs [42]. On the one hand, it inhibits the expression of proinflammatory cytokines such as IL-25, IL-8 and chemokines such as IFN-γ-induced CCL5 and CXCL10 in LECs [42,[44], [45], [46]]. The underlying mechanism might be that IL-22 can promote anti-microbial protein RegIIIγ production by LECs via STAT3 activation. RegIIIγ could act as a downstream effector molecule of IL-22 to reduce the production of proinflammatory molecules by LECs and attenuate the recruitment of eosinophils and ILC2s into the lung, thus significantly ameliorating airway hyperresponsiveness and allergic airway inflammation [42,47]. Furthermore, IFN-γ-induced MHC-I, MHC-II and CD54 upregulation on human bronchial epithelial (HBE) cells can be inhibited by IL-22, thereby protecting the lung epithelium against T cell-mediated cytotoxicity that can lead to chronic asthma [46]. These findings indicate that IL-22 might play critical anti-inflammatory roles in allergic asthma and other airway inflammation, providing a potential therapeutic strategy for these diseases. In combination with IL-17A, which can also be produced by ILC3s, IL-22 prominently promotes host defense genes such as *DEFB4A, S100A7* and *IL19* expression in HBE cells [48]. In addition, IL-22 increases clonogenic frequencies and migration of HBE cells, and enhances recovery of epithelial resistance in impaired epithelium [46,48]. These effects of IL-22 on HBE cells are critical to host defense against extracellular pathogens at mucosal sites and demonstrate the protective property of IL-22. On the other hand, it has also been found that IL-22 is essential for the onset of asthma during the sensitization phase as shown by the impaired eosinophil infiltration, decreased levels of inflammatory cytokines and chemokines, and mitigatory allergic airway inflammation in mice deficient in IL-22, but conversely possess protective functions during the effector phase as mentioned above, suggesting a dual role for IL-22 in allergic asthma [49]. Besides, IL-22 could increase granulocyte-colony stimulating factor (G-CSF) and the neutrophilic granulocyte-attracting chemokines CXCL1 and CXCL5 in respiratory epithelial cells, thus resulting in airway inflammation by recruiting and activating lymphocytes, also exhibiting the proinflammatory property [49]. Further investigation is warranted to fully appreciate the mechanism of crosstalk between ILC3s and LECs in mediating the distinct functions of IL-22 in airway inflammation and other lung diseases.

Although all three ILC subsets have been characterized in the lung, most investigations to date have centered on the roles of ILC2s in lung diseases. However, the ability of ILC3s to rapidly release IL-17A and IL-22 implicates an intriguing area for future investigation on the relationship between ILC3s and lung health.

### 2.4 ILC3s and other epithelial cells

ILC3s are the major ILC subset in fetal thymus, but are almost undetectable after birth [50]. Thymic epithelial cells (TECs) can be divided into cortical TECs and medullary TECs, according to their distinct locations and functions in the thymus [51]. IL-22 production by ILC3s, which is regulated by DC-derived IL-23, can induce TEC regeneration through IL-22R after thymic injury [51,52]. Importantly, the ILC3-IL-22-TEC axis is conducive to immune reconstitution after allogeneic hematopoietic transplantation [52]. Moreover, RANK signals from intrathymic CD4^+^CD3^−^RANKL^+^ LTi mediate the development of CD80^−^Aire^−^ medullary TEC progenitors into CD80^+^Aire^+^ medullary TECs, and LTi-expressed LTα is also associated with the maturation and organization of TECs [53]. In detail, RANK ligand (RANKL) is upregulated in LTi cells at the early phase of thymic regeneration and then induces LTα1β2 expression by LTi, which is critical to TEC regeneration upon bone marrow transplantation [54].

In addition, ILC3s also reside in the bladder and play an important role in mediating early defense against uropathogenic *Escherichia coli* (UPEC) infection [55]. Bladder epithelial cells can induce IL-17A production in bladder ILC3s during urinary tract infection through the release of IL-1β, and IL-17A subsequently mediates neutrophil recruitment to confer protection against UPEC [55].

## 3 Crosstalk between ILC3s and myeloid cells

ILC3s have complex dialogues with myeloid cells mostly through the production and reception of cytokines. On the one hand, ILC3s depend on the cytokines generated by myeloid cells, which can directly respond to microbial signals, for their activation and effector function [56]. On the other hand, ILC3s also act as an important regulator of the generation and activity of myeloid cells. Consequently, there exist complex loops within the interactions between ILC3s and myeloid cells, which play vital roles in defending against or conversely driving diseases. In the following text, we summarize crosstalk between ILC3s and representative myeloid cells: macrophages, neutrophils and DCs.

### 3.1 ILC3s and macrophages

Macrophages can respond to a wide range of microbial products, and play an important role in maintaining intestinal lymphocyte homeostasis. CCR2^+^ monocytes preferentially differentiate into CD11b^+^CD11c^+^F4/80^+^CD103^−^ macrophages (MP1) after they are recruited to the sites of infection, where MP1-derived IL-1β and IL-23 facilitate IL-22 production by intestinal ILC3s to prevent *C. rodentium* infection and infectious colitis in both human and mice [57,58]. IL-1β secreted by macrophages can also induce granulocyte-macrophage colony-stimulating factor (GM-CSF) production by ILC3s in a myeloid differentiation primary response gene 88 (Myd88)-dependent way, while IL-23 cannot [59]. Moreover, macrophage-expressed β2-integrin can also promote IL-22 production by ILC3s via Rac1-mediated induction of reactive oxygen species, which further upregulates IL-1β production via caspase-11-mediated non-canonical NLRP3 inflammasome activation in respons*e to C. rodentium* [60]. In addition, a recent study found that *Candida albicans* facilitate IL-7 generation through upregulating HIF-1-dependent glycolysis in macrophages [61]. IL-7, alone or synergizing with IL-1β or IL-23, can also increase IL-22 production in ILC3s via aryl hydrocarbon receptor (AhR) and STAT3, and eventually promote colitis-associated colon cancer progression [61].

ILC3s can also regulate the activity of macrophages. In the context of intestinal infection and inflammation, ILC3-derived GM-CSF facilitates the maturation and polarization of inflammatory intestinal macrophages, especially inducing the anti-microbial inflammatory M1 macrophages to defend against enteropathogenic infection but potentially cause pathological inflammation, while restraining a tissue-reparative macrophage phenotype to limit the process of intestinal fibrosis and stricturing as a serious complication in IBD by decreasing its production of collagen and platelet-derived growth factor [62]. Critically, GM-CSF-mediated macrophage polarization and accumulation in turn augment the production of cytokines that contribute to activating ILC3s and Th17 cells by macrophages, consequently forming a positive feedback loop to promote the function of ILC3s [58,62]. Moreover, ILC3-produced GM-CSF is essential to the maintenance of the function of macrophages to promote colonic regulatory T (Treg) cell homeostasis [59].

Together, it's clear that cytokines like IL-1β, IL-23 and GM-CSF play significant roles in the crosstalk between ILC3s and macrophages to protect against pathogens and maintain lymphocyte homeostasis.

### 3.2 ILC3s and neutrophils

Neutrophils are critical to the elimination of pathogens and damaged tissue and cells, and have a central position in acute inflammation [63,64]. Aside from their proinflammatory characteristics, they also have a role in regulating angiogenesis, tissue repair and immune responses [65]. Like macrophages, neutrophils can also be activated and regulated by ILC3-derived GM-CSF [66]. In particular, GM-CSF can promote neutrophil survival and induce their expression of peculiar immunoregulatory factors (HB-EGF and IL-1 receptor antagonist), which are associated with angiogenesis and blastocyst implantation in decidua contributing to a successful pregnancy [65,67]. Upon the stimulation of intestinal microbiota, ILC3s could produce IL-17A, which further increases plasma G-CSF concentration and the numbers of circulating and bone marrow neutrophils [68]. Moreover, ILC3s may influence neutrophil migration by producing CXCL8, which acts as a potent neutrophil chemotactic factor [65]. Therefore, ILC3s could indirectly regulate granulocytosis, neutrophil homeostasis and host resistance to sepsis [68]. Conversely, it has been found that recombinant IL-22 treatment or adoptive transfer of ILC3s could suppress neutrophil accumulation near alveoli and reduce the generation of neutrophil elastase 2 by neutrophils, thus repressing excess inflammation and preventing epithelial cell damage in the lungs of type 2 diabetes mellitus mice infected with *Mycobacterium tuberculosis* [69]. In contrast, a previous study found that IL-22 promotes neutrophil recruitment to the colon after colitis induction [70], indicating that the effect of IL-22 on neutrophils may be specific to different tissue microenvironments.

ILC3s are also regulated by neutrophils. A recent study found that apoptotic neutrophil-derived lysophosphatidylserine directly facilitates ILC3 activation and IL-22 production during experimental colitis [71]. In detail, dying neutrophils act as a signal of tissue damage, which can be recognized by ILC3-expressed G protein-coupled receptor (GPR) 34 and then promotes IL-22 production to facilitate IEC proliferation and trigger tissue repair [71]. In addition to lysophosphatidylserine, acetate-mediated IL-1β production by neutrophils can also activate ILC3s and induce IL-22 production, which is crucial to the protection against *Clostridium difficile* infection [72].

Collectively, these findings cast light on a potential and complex circulation between ILC3s and neutrophils, which plays an important role in maintaining tissue homeostasis or conversely, driving pathogenesis.

### 3.3 ILC3s and dendritic cells

DCs can induce both innate and adaptive immune responses upon infection. Like macrophages, TLR5-expressing CD103^+^CD11b^+^ lamina propria DCs can produce IL-23, in stimulation with the bacterial protein flagellin that activates TLR5, to upregulate IL-22 expression by ILC3s and subsequently initiate antimicrobial defense through inducing RegIIIγ production by IECs [73]. Compared with CX3CR1^+^ mononuclear phagocytes (MNPs), conventional CD103^+^ DCs produce less IL-23 and are less efficient in promoting IL-22 production in ILC3s [57]. ILC3-derived LT can activate LTβR on DCs to promote IL-23 production, which in turn triggers IL-22 production by ILC3s, consequently forming a positive feedback loop for control of *C. rodentium* infection [74]. Besides IL-23, the activation of Myd88 signaling in DCs can also induce the production of IL-22 by ILC3s to defend against *C. rodentium* invasion [75]*.* Moreover, DC-generated CXCL16 is critical to maintaining homeostasis, mediating migration and localization, and triggering IL-23-inducible IL-22 production of intestinal NKp46^+^ ILC3s, which highly express chemokine receptor CXCR6 [76]. Notably, CXCL16/CXCR6 interactions contribute to maintaining mucosal barrier function under steady-state conditions and in response to pathogens [76]. In addition to IL-22, IL-17A production by ILC3s can also be significantly promoted by CD103^−^CD11b^+^ colonic DC-derived TNF-α in combination with IL-23 and contributes to TRUC IBD as mentioned above [20]. Furthermore, in the intestinal lamina propria, DCs can regulate the plasticity of CD127^+^ ILC1s and ILC3s. It has been found that CD14^+^ DCs can facilitate the conversion from ILC3 to CD127^+^ ILC1, which may be associated with Crohn's disease displaying a decreased frequency of ILC3s but significantly expanded ILC1s, while CD103^+^ DCs can accelerate the differentiation of CD127^+^ ILC1 to ILC3 by producing retinoic acid (RA) and thus promote IL-22 production [77].

A transcriptionally distinct subset of intestinal conventional DCs (cDCs) has been found and termed CIA-DCs. They highly express IL-22BP that curtails the availability of bioactive IL-22 and increases the expression of lipid transporter by IECs, thus promoting the intestinal absorption and systemic metabolism of lipids [78]. Importantly, differentiation of CIA-DCs from another subset cDC2 requires programming by CCR6^+^ ILC3s via direct LTα1β2/LTβR signaling from ILC3s to CIA-DCs [78]. Therefore, LT/LTβR is critical to both ontogeny and IL-23 production of DCs mediated by ILC3s.

## 4 Crosstalk between ILC3s and adaptive immunity

Recent studies have revealed that ILC3s produce various factors that functionally regulate adaptive immunity in direct or indirect manners, and thus play vital roles in bridging innate and adaptive immune system. In turn, adaptive immunity reciprocally exerts effects on ILC3 survival and functions. Here, we summarize our current understanding of the functional crosstalk between ILC3s and the adaptive immune system.

### 4.1 Regulation of adaptive immunity by ILC3s

LTi/LTi-like cells play a critical role in inducing lymphoid tissue organogenesis during fetal development as well as the establishment of tertiary lymphoid structures like isolated lymphoid follicles (ILFs) and cryptopatches after birth [79]. In detail, the formation of secondary lymphoid organs before birth depends on the interaction between LTi cells that produce LTα_1_β_2_ and stromal organizer cells that express LTβR, and the interaction between RORγt^+^ LTi cells and stromal cells can induce the recruitment of B cells and DCs to the cryptopatches to form ILFs [80,81]. Moreover, the IL-7-dependent development of LTi is essential for T and B cells homing to lymph nodes [82]. In addition, both T-dependent and independent IgA production require ILC3s. RORγt^+^ ILC-derived soluble lymphotoxin α (sLTα3) induces T cell-dependent production of IgA in the lamina propria via control of T cells homing to the gut, while LTα_1_β_2_ on ILC3s controls inducible nitric oxide synthase expression by DCs, which subsequently induce TGFβ activation via integrin αvβ8 during interactions with B cells and consequently support T cell-independent IgA production [83,84].

In addition to inducing lymphoid organogenesis and IgA production, ILC3s are also crucial to the regulation of T and B cells directly through secretion of functional cytokines and presentation of antigens, or indirectly via interactions with innate immune cells, epithelial cells and microbiota.

**Fig. 2:**
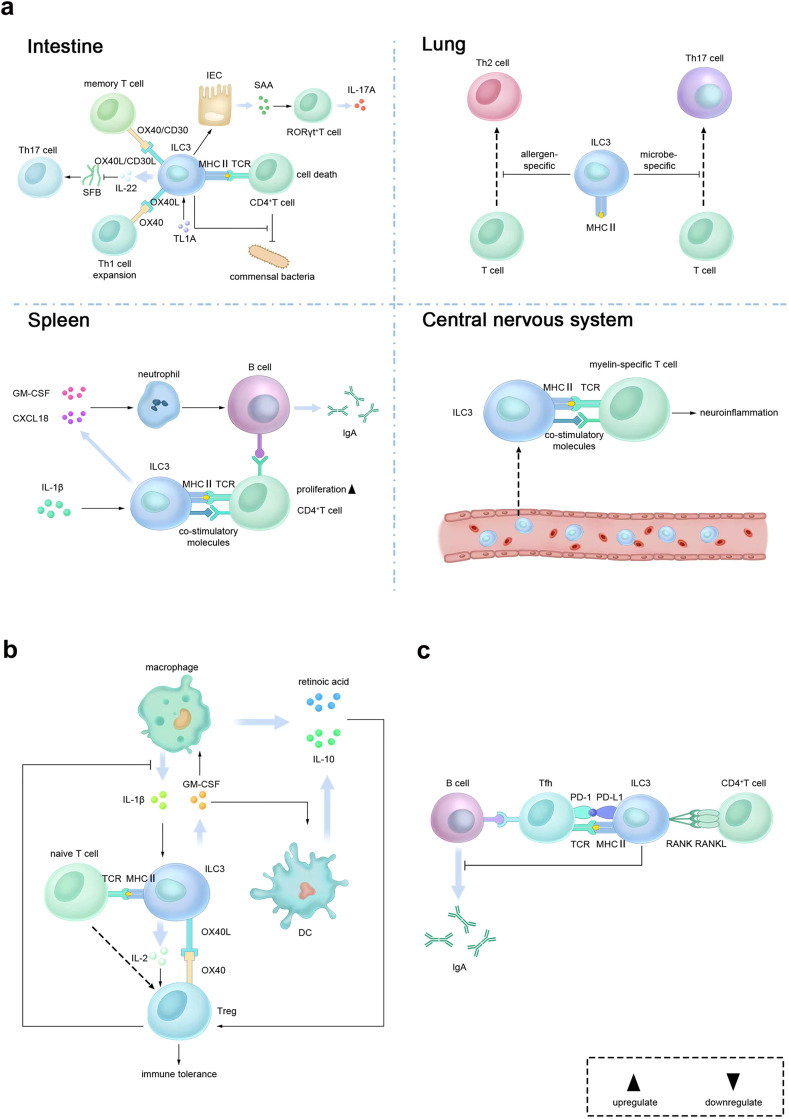
**Regulation of adaptive immunity by ILC3s.** (a) Regulation of adaptive immune responses by ILC3s in different tissues. In the intestine, ILC3s can directly or indirectly regulate CD4^+^ T cell through MHC-II, OX40L, CD30L and IL-22. In lung-draining lymph nodes, MHC-II^+^ ILC3s can limit the responses of Th2 and Th17 cells to allergens and microbial antigens. However, in the spleen and central nervous system, MHC-II^+^ ILC3s can promote CD4^+^ T cell responses. Additionally, ILC3s indirectly promote B cell response and IgA production in the spleen. (b) Regulation of Treg cells by ILC3s. ILC3s can directly stimulate the differentiation of Treg cells from naive T cells via MHC-II-dependent antigen presentation, and maintain Treg homeostasis through IL-2 and OX40L signaling. ILC3s can also indirectly promote Treg cells through GM-CSF-dependent regulation of macrophage. Interestingly, Treg cells can suppress IL-1β secretion from CX3CR1^+^ macrophages and thus negatively regulate ILC3s. (c) Regulation of Tfh cells by ILC3s. ILC3s can inhibit B cell responses through regulating Tfh cells in a MHC-II and PD-L1-dependent manner. Abbreviations: ILC3, group 3 innate lymphoid cell; Treg, regulatory T; OX40L, OX40 ligand; DCs, dendritic cells; IL, interleukin; GM-CSF, granulocyte-macrophage colony-stimulating factor; MHCII, major histocompatibility complex class II; TL1A, TNF-like ligand 1A; SFB, segmented filamentous bacteria; Th, T helper; SAA, serum amyloid A; IECs, intestinal epithelial cells; RANKL, RANK ligand; Tfh, T follicular helper; mLN, mesenteric lymph node.

A significant conceptual progress that associates ILC3s with the adaptive immune system was the finding that ILC3s can express major histocompatibility complex class II (MHCII), process and present antigens and directly regulate the adaptive immunity. Antigen presentation by ILC3s has been shown critical and sufficient to mediating microbiota-specific differentiation of Treg cells and suppressing the expansion of pathogenic Th17 cells, thereby building immune tolerance to microbiota in the intestine and preventing aberrant inflammation [85,86]. Besides antigen presentation, it is increasingly appreciated that ILC3-derived TNFR-family ligand OX40L is critical to the homeostasis of Treg cells through OX40L-OX40 interaction [87,88]. In addition to the direct regulation, myeloid cells mentioned above can function as hubs between ILC3s and Treg cells. ILC3s are the primary cellular source of IL-2, which is promoted by macrophage-derived IL-1β in response to intestinal microbiota and is critical to the maintenance of Treg cells and tissue homeostasis in the small intestine [89]. Macrophage-derived IL-1β can also increase the secretion of GM-CSF from ILC3s, which reciprocally induce RA and IL-10 production by DCs and macrophages, contributing to the maintenance of intestinal Treg homeostasis and oral tolerance to dietary antigens [59] (Fig. 2b).

In addition to the regulations of Treg cells for establishing immune homeostasis, ILC3s also have direct or indirect effects on adaptive immune responses (Fig. 2a). In the intestine, ILC3s can induce CD4^+^ T cell death through antigen presentation and limit their excessive inflammatory responses to commensal bacteria in a MHCII-dependent way, thereby protecting host against varieties of chronic human diseases like intestinal inflammation [90,91]. Notably, in the context of colon cancer, the crosstalk between ILC3s and T cells via MHCII plays an important role in maintaining colonization of microbiota that promotes type-1 immunity, and thus decreases the susceptibility to invasive and flat colon tumor development [92]. Splenic MHCII^+^ ILC3s can promote CD4^+^ T cells proliferation as well as T cell-dependent B cell responses through upregulation of the expression of MHCII and co-stimulatory molecules upon stimulation with IL-1β [93], while MHCII^+^ ILC3s enriched in the lung-draining lymph nodes of healthy mice and human can limit Th2 cell responses to allergens and Th17 cell responses to microbial antigens in the airway [94]. Moreover, in the central nervous system, a subset of ILC3s derived from the circulation have been found recently to promote myelin-specific T cell responses via antigen presentation during neuroinflammation, while peripheral and tissue-resident ILC3s that are experimentally targeted to present myelin antigens can eliminate autoimmune T cells, mediate tolerance and protect against neuroinflammation [95]. Besides MHCII, ILC3-expressed OX40L and CD30L also play important roles in regulating the adaptive immune response. LTi are essential for the persistence of memory CD4^+^ T cells within the lamina propria through OX40-OX40L and CD30-CD30L interactions [96,97]. Furthermore, CX3CR1^+^ MNP-secreted TNF-like ligand 1A (TL1A) in response to IBD-associated microbiota promotes OX40L expression on MHCII^+^ ILC3s, which further co-stimulates pathogenic Th1 cell expansion and consequently induces chronic T cell colitis [87]. Apart from direct regulation, ILC3-derived IL-22 can also mediate the crosstalk between ILC3s and T cells via microbiota and IECs. It has been found that IL-22-mediated downregulation of microflora such as segmented filamentous bacteria (SFB) is related to impairment of systemic aberrant Th17 responses and development which are involved in the pathogenesis of autoimmune diseases like IBD, rheumatoid arthritis and psoriasis [98,99]. Conversely, in addition to the inhibitory effects on T cells, IL-22 can also promote the production of epithelial serum amyloid A proteins by IECs, which has the ability to directly increase local IL-17A expression in RORγt^+^ T cells in the terminal ileum [100].

ILC3s can also interact with T follicular helper (Tfh) cells. Through presenting antigens to Tfh cells, ILC3s in the interfollicular regions of the intestinal draining lymph nodes can inhibit colonic IgA generation in response to commensal and pathogenic microbiota [101] (Fig. 2c).

Moreover, as for B cells, splenic NCR^+^ ILC3s express B cell-activating factor (BAFF), the ligand of the costimulatory receptor CD40 (CD40L) and the Notch2 ligand Delta-like 1 (DLL1) on the surface and secrete BAFF, thus facilitating the survival, proliferation, IgM production and plasmablast differentiation of marginal zone (MZ) B cells [66]. Accordingly, ILC3-derived BAFF has been found to promote IL-15 generation by B cells [102]. IL-15 in turn increases CD40L expression on ILC3s and CD40L^+^ ILC3s have similar effects to those mentioned above on B cells, which may partially explain the mechanisms behind those impacts [102]. In the spleen, ILC3s also express GM-CSF and CXCL18, which play critical roles in the activation, survival and recruitment of neutrophils equipped with B cell-helper function, stimulating IgA production in MZ B cells [66].

Therefore, ILC3s seem to have different effects on the adaptive immune system in distinct tissues. In detail, it can maintain Treg in homeostasis as well as activate immune responses to drive inflammation. This heterogeneity has been demonstrated to be associated with different regulations by the transcriptional regulator ID2 in different microenvironments [103].

### 4.2 Regulation of ILC3s by adaptive immunity

We have discussed the direct and indirect regulation of adaptive immunity by ILC3s. Reciprocally, it has now emerged that the adaptive immunity also modulates ILC3 activity.

Studies have shown that the production of IL-22 by ILC3s, the absolute number and proliferative ability of ILC3s and ILC3-induced antimicrobial peptides expression by IECs are all higher in mice lacking RAG-1 or RAG-2, which indicates potential repression of ILC3 survival and function conferred by adaptive immunity [24,104]. In particular, CD4^+^ T cells play a key role in the inhibitory effects through antigen-specific T cell receptor signals and repression of STAT3 phosphorylation in ILC3s and IECs [104,105]. Among CD4^+^ T cells, Treg cells negatively regulate ILC3s by reducing IL-23 and IL-1β secretion from CX3CR1^+^ macrophages (Fig. 2b), while Th17 cells do so by limiting the abundance of SFB [105,106]. Importantly, the suppression of ILC3s by Treg cells contributes to repressing ILC3-mediated colitis, indicating that promoting the function of Treg cells or limiting the activity of CX3CR1^+^ macrophages would be a promising therapeutic option for treatment of IBD [106].

Converse to the inhibitory effects on ILC3s exerted by CD4^+^ cells, we also found that RANKL signaling from CD4^+^ T cells is indispensable for the maintenance of LTi-like cells in the mesenteric lymph node (mLN), which could in turn suppress the development of Tfh cells and the humoral responses in an ID2- and PD-L1-dependent way, acting as potential gatekeepers for appropriate humoral responses to immunostimulants from the intestine [103] (Fig. 2c).

## 5 Crosstalk between ILC3s and other cells

In addition to immune and epithelial cells, ILC3s also have complex interplay with other cells such as the neurons, glial cells, stromal cells and so on. Here we discuss the regulation of ILC3s by the nervous system and bidirectional interactions between ILC3s and stromal cells.

### 5.1 The neuro-regulation of ILC3s

The neural reflex can sense peripheral inflammation and has a role in mediating resolution of tissue and infectious inflammation [107]. Notably, ILC3s have been found to selectively express various neurotransmitter receptors and neuropeptide receptors, demonstrating the potential association between ILC3s and nervous system [108]. The interactions between ILC3s and acetylcholine (ACh)-secreting vagal system,vasoactive intestinal peptide (VIP) neurons and glial cells are illustrated as follows.

The regulation of ILC3s during inflammation by the vagus is partially mediated via the secretion of ACh and it has been found that disruption of the vagal system results in significantly decreased numbers of ILC3s, thereby delaying the resolution of *E. coli*-induced bacterial infection [107]. In particular, exposure of ILC3s to ACh increases 17‑hydroxy-4Z,7Z,10Z,13Z,15E,19Z-docosa-hexaenoic acid, which acts as the biosynthetic pathway marker in the host-protective mediator protectin conjugates in tissue regeneration 1 (PTCR1) metabolome in macrophages, and macrophage-derived PCTR1 functions as a pro-resolving circuit and has the ability to restore the dysregulated inflammatory response in vagotomized mice [107].

Furthermore, ILC3s also express VIP receptor type 2 (VIPR2), which mediates the inhibition of IL-22 production in ILC3s in response to food intake, thereby repressing the antimicrobial peptide RegIIIγ secretion from IECs but upregulating lipid-binding proteins and transporters [108]. Therefore, the effect of enteric VIPergic neurons on ILC3s allows the growth of SFB and promotes nutrient absorption during food consumption [108]. Importantly, this mechanism provides an unprecedented opportunity to therapeutically harness VIPR2 inhibitors to suppress bacteria proliferation during infections.

In addition to VIPR2, ILC3s have also been found to highly express the neuroregulatory receptor RET, through which neurotrophic factors from enteric glial cells mediate the control of IL-22 production by ILC3s and intestinal epithelial reactivity [109]. This mechanism demonstrates that glial cells are central sentinels for neuronal and innate immune regulation.

### 5.2 ILC3s and stromal cells

Stromal cells have long been considered to simply provide structural support to the lymphoid organs and create different microdomains for T and B cells [110]. However, extensive research has focused on the interaction of stromal cells with immune cells. For example, secondary lymphoid tissue organogenesis as well as T and B lymphopoiesis require crosstalk between LTi and stromal cells through LT-LTβR, RANK-RANKL, IL-7-IL-7R, CXCL13-CXCR5 and other interactions [80,111,112]. Here we discuss the interplay of mesenchymal stromal cells (MSCs), intestinal stromal cells and marginal reticular cells (MRCs) with ILC3s.

The interaction between MSCs and ILC3s is largely mediated through cell-cell contact [113]. MSCs can promote the proliferation and IL-22 production of ILC3s, potentially involved in the protection against tissue damage induced by GVHD and other inflammatory diseases, and reciprocally ILC3s increase ICAM-1 and VCAM-1 expression on MSCs [113].

The crosstalk between ILC3s and intestinal stromal cells plays an important role in regulating the accumulation, distribution and anti-microbial and tissue-protective functions of ILC3s. ILC3s highly express GPR183, which mediates their migration towards its ligand 7α,25-OHC produced by intestinal stromal cells [114]. Through binding to 7α,25-OHC produced by intestinal stromal cells, GPR183 controls the distribution and accumulation of ILC3s in the mLNs, Peyer's patches and intestine. As a consequence, GPR183 and 7α,25-OHC promotes ILC3-mediated protective immunity against bacterial infection by increasing the number of IL-22-producing ILC3s in the intestine [114].

MRCs are a MZ subset of stromal cells, maintaining the survival of ILC3s via contact-dependent interactions and contact-independent signals, including ILC survival cytokines such as IL-7, IL-23 and IL-1β, and ILC-recruiting chemokines like CCL20 [66]. Conversely, ILC3s can promote expression of IL-7, CCL20 and MAdCAM-1 in MRCs via LT and TNF, which indicates potential positive loops within the bidirectional crosstalk between ILC3s and stromal MRCs [66].

## 6 Conclusions and perspectives

ILC3s are a heterogeneous group of the ILC family that is abundantly localized in mucous membranes and attributed essential roles in orchestrating tissue homeostasis and immune defense. Here we discuss the interplay between ILC3s and immune or non-hematopoietic cells, thereby highlighting the central role of ILC3s as vital hubs in different tissue regulatory circuits, in which ILC3s receive and interpret various signals derived from multiple physiological systems, and express functional factors like IL-22, IL-17, LT and co-stimulatory molecules to modulate downstream effector cells and reactions in the context of health and disease [115]. Given the complexity of the crosstalk between ILC3s and other cells, how ILC3s coordinate the signals of varying sources and natures to exhibit final effects requires future investigation. The potential crosstalk and convergence among signaling pathways downstream of receptors of functional molecules sensed by ILC3s may play vital roles in modulating the property and magnitude of ILC3 responses. For instance, STAT3, the downstream adaptor of IL-23 receptor signaling in ILC3s has been shown indispensable for IL-22 production to protect against *C.rodentium* infection [116]. STAT3 could be recruited to the *Il22* locus and thus induce IL-22 production at the transcriptional level. Furthermore, progress in single-cell RNA sequencing and epigenetic profiling will greatly help uncover the signaling transduction and transcriptional dynamics behind the integration of various cues and synchronization of immune responses by ILC3s [117].

There are also many details remaining to be clarified in the future investigations. Some of the existing findings fail to adopt unified classification standard established in 2013, thus making it difficult and inaccurate to link them with each other, as the functional ILC3s in these articles may not be from the same type [1]. Besides, the cellular sources of critical cytokines that play important roles in intercellular communications, such as IL-22 mentioned above, should be confirmed, for these molecules may come primarily from different cells under different conditions like distinct diseases or distinct phases of the same disease. For instance, it has been found that IL-17A could regulate the tight junction protein occludin of IECs and maintain epithelial barrier integrity [118]. However, although ILC3s can also generate IL-17A, the study pointed out that γδ T cells rather than ILC3s were the predominant source of IL-17A in the lamina propria after DSS-induced injury and thus contributed more to maintaining epithelial integrity [118]. Therefore, it is necessary to rethink and update all findings when new results about details listed above come out.

Moreover, many inconsistent results mentioned above indicate that the effects of the interactions between ILC3s and other cells could be different in distinct microenvironments or influenced by other factors. For example, as mentioned above, ILC3-generated sLTα3 could induce production of IgA in the lamina propria, whereas ILC3s in the interfollicular regions of the intestinal draining lymph nodes inhibit colonic IgA generation [83,101]. The potential influence factors and mechanisms require further exploration.

As for the interactions between ILC3s and the adaptive immune system, most recent studies focus on the crosstalk between ILC3s and T cells, especially CD4^+^ T cells. Therefore, future studies are warranted to pay more attention to the interactions between ILC3s and B cells or other types of T cells. Also, when it comes to the interplay between ILC3s and epithelial cells, the potential mechanisms of balancing the protective and proinflammatory effects of ILC3s on epithelial cells and the potential symptoms caused by the imbalance remain an additional area of investigation.

In the future, it is expected that additional mechanisms of cellular interactions between ILC3s and other cells will be discovered, which may contribute to a deeper and broader appreciation of relevant diseases and provide more available targets for therapeutic approach.

## Declaration of competing interest

The authors declare that they have no conflicts of interest in this work.
